# Prevalence of enteric bacteria and their antimicrobial susceptibility patterns among food handlers in Gondar town, Northwest Ethiopia

**DOI:** 10.1186/s13756-019-0566-7

**Published:** 2019-07-08

**Authors:** Michael Getie, Wondwossen Abebe, Belay Tessema

**Affiliations:** 1 Department of Laboratory, University of Gondar Comprehensive Specialized Hospital, Gondar, Ethiopia; 2 Department of Medical Microbiology, School of Biomedical and Laboratory Sciences, College of Medicine and Health Sciences, University of Gondar, P.O.Box:196, Gondar, Ethiopia

**Keywords:** Food handlers, Enteric bacteria, Antibiotic susceptibility

## Abstract

**Background:**

Enteric bacterial pathogens are the major causes of food-borne gastroenteritis in humans and remain important public health problems worldwide. The emergence of antimicrobial resistance is a global concern, particularly in developing countries. The aim of this study was to determine the prevalence of enteric bacteria pathogens and their antimicrobial susceptibility patterns among food handlers in Gondar town, Northwest Ethiopia.

**Methods:**

A cross-sectional study was conducted from February 4 to April 16, 2018. A total of 257 food handlers were selected using a multistage sampling technique. Data on socio-demographic characteristics were collected using a structured questionnaire. Stool samples were collected and inoculated into appropriate media. Enteric bacterial pathogens were identified using standard microbiological methods. Antimicrobial susceptibility tests were performed using the disk diffusion technique as per the standard Kirby-Bauer method. Data were entered and analyzed using SPSS version 20 software.

**Results:**

The overall prevalence of enteric bacteria was 34/257 (13. 2%, [95% CI, 8.9–17.5%]). *Shigella* species was the leading isolate that accounted for 26/257 (10.1%) followed by Enterohemorrhagic *Escherichia coli* (EHEC) O157: H7 5/257 (1.9%) and *Salmonella* species 3/257 (1.2%). *Shigella* spp. was susceptible to ciprofloxacin 26 (100%), ceftriaxone 25 (96.1%), chloramphenicol 24 (92.3%), nalidixic acid 24 (92.3%), and gentamicin 20 (76.9%). *Escherichia coli* O157: H7 and *Salmonella* spp. showed the maximum (100%) susceptibility results to ceftriaxone, chloramphenicol, ciprofloxacin, and gentamicin. The overall prevalence of Multidrug resistance (MDR) in the current study was 14/34 (41.2%).

**Conclusion:**

Our study showed high prevalence of enteric bacterial pathogens among food handlers. All isolates were susceptible to ciprofloxacin. However, a substential number of isolates were resistant to commonly prescribed antibiotics and the prevalence of MDR was high.

## Background

World health organization, food borne disease burden epidemiology reference group estimated that 31 food borne diseases (FBD) resulted in over 600 million illnesses and 420,000 deaths in 2010 globally. However, there were considerable differences in the burden of food borne diseases among sub regions delimited on the basis of child and adult mortality. The highest burden per population was observed in Africa [1]. It is estimated that more than 200 types of diseases transmitted to human through the ingestion of food contaminated with microorganisms or with chemicals. Food contamination can occur at any stage of food production, food processing, food storage and preparation in restaurant and hotels. Contamination can arise because of the pollution of the water, soil or air or through poor food-handling practices such as failing to wash one’s hands before preparing food [2].

A wide variety of etiological agents are responsible for causing food borne diseases, the most prominent emerging problems are of microbial origin responsible for 79% of the FBD burden. The most significant pathogens are *Salmonella* spp., toxigenic *Escherichia coli*, Norovirus and *Campylobacter* [1, 2].

The high burden of infectious diseases and inappropriate use of antibiotics to humans, animals, and agriculture contribute for the emergence of antimicrobial resistance globally, particularly in developing countries [3]. The previous studies have shown an increasing incidence of multidrug resistance in food borne enteric pathogens, particularly to the commonly used antimicrobial agents such as ampicillin, chloramphenicol, streptomycin, sulphadiazine and tetracycline [3, 4].

Most of the studies conducted in Ethiopia were institution based, for instance a hospital-based Summary report by the Ministry of Health revealed that the annual incidence of food-borne illnesses ranged from 3.4 to 9.3%, the median is 5.8% [5]. Furthermore, studies conducted in different parts of the country documented that *Salmonella spp*, *Shigella spp*, *Escherichia coli*, and *Staphylococcus aureus* were the most common isolates from food handlers with a high rate of drug resistance to the commonly used antibiotics [6–8]. However, there are limited data in Gondar town. Therefore, the aim of this study was to determine the prevalence of enteric bacterial pathogens and their antimicrobial susceptibility patterns among food handlers working in Gondar town restaurants and hotels, Northwest Ethiopia.

## Methods

### Study design, area and population

A cross-sectional study was conducted from February to April 2018 among food handlers working at food establishments found in Gondar town. The town is located 747 km Northwest of Addis Ababa and 182 km far from BahirDar, the capital city of the Amhara regional state. According to the Federal Democratic Republic of Ethiopia Central Statistical Agency of Population projection values of 2017, the total population of the town is 360,600 (176,593 males and 184,007 females) [9]. It has 13 sub-cities which consist of 25 kebeles and various food establishments such as hotels and restaurants.

### Sample size and sampling technique

The minimum sample size was calculated based on the assumption of 5% expected margins of error and 95% confidence interval, taking the prevalence of enteric bacteria (8.7%) from the previous study which was conducted in WolaitaSodo town [8] using a single population proportion formula. Since the total number of the source population was less than 10,000, correction formula was used to adjust and it gives 95. Finally, by considering a 10% non-response rate and design effect of 2, the final minimum sample size was 210 study participants. The study participants were sampled by using a multistage sampling technique. The primary procedure was selecting the sub-cities followed by selection of the catering establishments. Five sub-cities were selected by simple random sampling methods using lottery methods among the existing 13 administrative sub-cities of Gondar town. Subsequently, a comprehensive list of existing catering establishment was obtained from the Gondar trade administration office and the entire catering establishment list within those selected sub cities was stratified as hotels and restaurants. A proportion sampling technique was used to determine for each stratum and the selection was performed using simple random sampling by lottery methods.

### Data collection

Socio-demographic data was gathered using a structured pre-tested questionnaire. The questionnaire included information on sex, age, educational status, and service year among others.

### Specimen collection, culture and identification

Stool samples were collected using clean, dry and leakproof stool cups and immediately placed into Cary-Blair transport medium (Oxoid Ltd., Basingstoke, UK). Samples were transported to University of Gondar Medical Microbiology Laboratory in cold box with ice-packs within 2 h of collection for further processing. Stool samples were directly inoculated onto MacConkey agar, Salmonella-Shigella agar and Xylose Lysine Deoxycholate agar after enrichment with Selenite cystine broth and incubated at 37 °C for 18–24 h. After incubation, isolates were characterized by following the standard biochemical test, including triple sugar iron, Hydrogen sulfide production, indole production and motility in sulfide-Indole-motility medium, citrate utilization, lysine decarboxylase in simmon’s citrate agar, and urease production [10]. The *E.coli* isolates were also sub-cultured onto Sorbitol MacConkey agar to identify Enterohemorrhagic *Escherichia coli* (EHEC) and colorless colonies (nonsorbitol fermenter) were serologically confirmed using IGM antibodies to *E. coli* O157:H7 [8]. All the biochemical media were obtained from Oxoid Ltd., (Basingstoke, UK).

### Antimicrobial susceptibility testing

The antibiotic susceptibility testing of all strains were carried out on Muller-Hinton agar (Oxoid, UK) with antibiotic discs (Oxoid, UK) using Kirby-Bauer disc diffusion technique against amoxicillin (30 μg), ampicillin (10 μg), chloramphenicol (30 μg), ciprofloxacin (5 μg), ceftriaxone (30 μg), nalidixic acid (30 μg), co-trimoxazole (trimethoprim-sulfamethoxazole) (25 μg), gentamicin (10 μg), and tetracycline (30 μg) based on the guidelines adapted from Clinical and Laboratory Standards Institute (CLSI 2017 edition), the results were reported as sensitive, intermediate and resistance [11]. For statistical analysis, all isolates with intermediate reactions were classified as resistant. Multidrug resistance (MDR) is defined as the resistance of an isolate to three and more antimicrobial agents within one class of drug [7].

### Data analysis and interpretation

Data were entered into Epi-data 3.1 software and then exported to SPSS version 20 for analysis. Descriptive statistics such as median, inter quartile range, standard deviation, frequencies and percentages were computed. To check the association between dependent and independent variables, 5% level of precision was used.

## Results

### Socio-demographic characteristics

A total of 257 food handlers were included in this study. The majority of food handlers were females, 236 (91. 8%). The median ages and standard deviation of the study participant was 26 + 5.36 ranged from 17 to 47 years with an inter quartile range of 6. Eight five (33.1%) of them were between 27 and 30 years and 78 (30.4%) were between 23 and 26 years old. Most of the food handlers, 119 (46.3%) were served for a period of 1 to 5 years, 99 (38.5%) were completed secondary school and all the study participants were asymptomatic for diarrheal illnesses even though the study was designed to include both symptomatic and asymptomatic food handlers. The total of 163 (63.4%) studied establishments were restaurants (Table 1).

**Table 1:** Distribution of enteric bacteria isolated from food handlers in Gondar town, Northwest Ethiopia, 2018

Characteristic	*Shigella spp* (*n*, %)	EHEC* O157:H7 (*n*,%)	*Salmonella spp* (*n*, %)	Total (*n*,%)
Age in year				
< 22	5(1.9)	1(0.4)	1(0.4)	7(2.8)
23–26	13(5.1)	0(0)	1(0.4)	14(5.4)
27–30	7(2.7)	2(0.7)	1(0.4)	10(3.9)
> 31	1(0.4)	2(0.7)	0(0)	3(1.2)
Sub-total	26(10.1)	5(1.9)	3(1.2)	34(13.2)
Educational status				
Illiterate	6(2.3)	2(0.7)	0(0)	8(3.1)
Primary school	9(3.5)	1(0.4)	0(0)	10(3.9)
Secondary school	11(4.3)	1(0.4)	3(1.2)	15(5.8)
Diploma &above	0(0)	1(0.4)	0(0)	1(0.4)
Sub-total	26(10.1)	5(1.9)	3(1.2)	34(13.2)
Service year (in years)				
< 1	12(4.7)	3(1.2)	0(0)	15(5.8)
1–5	11(4.3)	1(0.4)	1(0.4)	13(5.1)
> 6	3(1.2)	1(0.4)	2(0.7)	6(2.3)
Sub-total	26(10.1)	5(1.9)	3(1.2)	34(13.2)
Place of work				
Hotel	7(2.7)	1(0.4)	0(0)	8(3.1)
Restaurant	19(7.4)	4(1.5)	3(1.2)	26(10.2)
Sub-total	26(10.1)	5(1.9)	3(1.2)	34(13.2)

EHEC*: Enterohemorrhagic *Escherichia coli*

### Prevalence of enteric bacteria

The overall prevalence of enteric bacteria was 34/257 (13. 2%, [95% CI, 8.9–17.5%]). *Shigella* species was the leading isolate that accounted for 26/257 (10.1%), of which, 3/257 (1.2%) isolates were *Shigella dysentery,* followed by EHEC O157:H7, 5/257 (1.9%) and *Salmonella* species, 3/257 (1.2%) (Table 1).

### Antimicrobial susceptibility pattern

The antimicrobial susceptibility pattern of enteric bacteria to different antibiotics is presented in Table 2. *Shigella* spp. isolates were susceptible to ciprofloxacin 26 (100%), ceftriaxone 25 (96.1%), chloramphenicol 24 (92.3%), nalidixic acid 24 (92.3%), and gentamicin 20 (76.9%). *Escherichia coli* O157: H7 isolates were 100% susceptible to ceftriaxone, chloramphenicol, ciprofloxacin, and gentamicin. *Salmonella* spp. isolates were 100% susceptible to ampicillin, ceftriaxone, chloramphenicol, ciprofloxacin and nalidixic acid, while 66.7% of the isolate were susceptible to gentamicin. Multi-Drug Resistance (MDR) was observed in 10 (38.5%) *Shigella* spp., of which, 7 (26.9%) isolates were resistant to more than five antimicrobials. The overall prevalence rate of MDR in this study was 14 (41.2%) (Table 3).

**Table 2:** Antimicrobial susceptibility pattern of enteric bacteria isolated from food handlers at Gondar town, Northwest Ethiopia, 2018

Bacterial Isolates		Antimicrobial susceptibility pattern (n,%)								
		AML (n,%)	AMP (n,%)	CRO (n,%)	CHL (n,%)	CIP (n,%)	SXT (n,%)	TTC (n,%)	NA (n,%)	GEN (n,%)
*Shigella* species (*n* = 26)	S	17(65.4)	10 (38.5)	25(96.1)	24 (92.3)	26 (100)	16 (61.5)	9 (34.6)	24 (92.3)	20(76.9)
	R	9 (34.6)	16 (61.5)	1 (3.9)	2 (7.7)	0	10 (38.5)	17 (65.4)	2 (7.7)	6(23.1)
EHEC* O157:H7(*n* = 5)	S	1 (20)	1 (20)	5 (100)	5 (100)	5 (100)	2 (66.7)	1 (20)	2 (40)	5(100)
	R	4 (80.)	4 (80)	0	0	0	1 (33.3)	4 (80)	3 (60)	0
*Salmonella* species(*n* = 3)	S	2 (66.7)	3 (100)	3 (100)	3 (100)	3 (100)	2 (66.7)	1 (33.3)	3 (100)	2(66.7)
	R	1 (33.3)	0	0	0	0	1 (33.3)	2 (66.7)	0	1(33.3)

Key: AML = Amoxicillin, AMP Ampicillin, CRO = Ceftriaxone, CHL = Chloramphenicol, CIP = Ciprofloxacin, SXT = trimethoprim-sulphamethoxazole, TTC = Tetracycline, NA = Nalidixic acid, GEN = Gentamicin, S = Sensitive R = Resistant, EHEC*: Enterohemorrhagic *Escherichia coli*

**Table 3:** Multidrug resistance patterns of pathogenic enteric bacteria isolated from food handlers at Gondar town, Northwest Ethiopia, 2018

Bacterial isolates	Degree of resistance			
	R3 n (%)	R4 n (%)	*R* *>* *5* n (%)	MDR** n (%)
*Shigella species*(n = 26)	3(11.5)	0	7(26.9)	10(38.4)
EHEC* O157: H7 (n = 5)	0	1(20)	2	3(60)
*Salmonella species*(n = 3)	0	1(33.3)	0	1(33.3)
Total (34)	3(8.8)	2(5.9)	9(26.5)	14(41.2)

EHEC*: Enterohemorrhagic Escherichia coli, **MDR = Isolates resistant to 3 or more antibiotics classes. R3 = Resistant to Three antibiotic classes, R4 = Resistant to four antibiotic classes, R *>* *5 = Resistant to greater or equal to 5 antibiotics*

## Discussion

Several studies have underlined that food handlers with poor personal hygiene could be potential sources of infection due to pathogenic bacteria [12–24]. The overall prevalence of entero-pathogenic bacteria isolates from stool in this study was (13.2%). This is consistent with the reported prevalence in a study done in South Ethiopia (10%) [12], Nigeria (17.2%) [13] and India (13.3%) [14]. However, it is higher than the reported prevalence by other studies in Gondar, Ethiopia (3.1%) [15], Addis Ababa, Ethiopia (3.5%) [16], Nigeria (6.9%) [17] and Jordan (7.4%) [18]. The observed differences in rates of bacterial isolation could be attributed to differences in food handling and santiation practice, study period and geographical varation [14, 15]. These asymptomatic food handler carriers could have continued working unaware of their status of infection by these food borne pathogens and keep transmitting the diseases to the community.

The isolation rate of *Shigella* species (10.1%) in our study was in agreement with the study conducted in India (9.3%) [14]. Conversely, this finding was lower than the results of a study conducted in Nigeria (15.5%) [19], and higher compared to similar studies conducted in Gondar, Ethiopia (3.1%) [15], Jimma, Ethiopia (0.9%) [7], Nigeria (2.2%) [17], and Iran (0.9%) [20]. This may indicate poor hygien, both personal and food handling practices and may lead to outbreaks of bacillary dysentery to the public [14]. In this study, EHEC (1.9%) was found to be in agreement with the study conducted in Ethiopia and India with the prevalence rate of 1.8 and 1.3%, respectively [7, 14]. However, this finding was higher than the study conducted in Kenya (0.1%) [21] and lower than the previous studies conducted in Ethiopia, Iran and Finland with a prevalence of 6.03, 34.5, and 9%, respectively [8, 22, 23]. The varying detection rates may be explained by differences in the study population and laboratory methods. The 1.9% EHEC positive food handlers in our study may serve as potential sources of outbreaks in the community.

In this study, the isolation rate of *Salmonella* (1.2%) was in agreement with the study conducted in Gondar, Ethiopia (1.08%) [15], Bahirdar, Ethiopia (1.4%) [24], India (1.4%) [14] and Ghana (2.3%) [25]. On the other hand, it was found to be lower than similar studies done in Ethiopia like Arba Minch (3.0%) [12], Jimma (4.1%) [7], Addis Ababa (3.5%) [16], and in Nigeria (17.2%) [13] and India (4.0%) [14]. The the isolation rate of *Salmonella* in the present study was higher compared to studies done in Iran, Jordan and Thailand, which report no isolation rate of *Salmonella* from the stools of food handlers [20, 26, 27]. This maight be due to epidomological diference and seasonal variation.

In this study *Shigella* spp. isolates were highly susceptible to ciprofloxacin (100%), ceftriaxone (96.1%), chloramphenicol (92.3%), nalidixic acid (92.3%), and gentamicin (76.9%) which is comparable with the reports from Eastern Ethiopia [28], and in South Ethiopia [12]. *Salmonella* spp. isolates showed high level of susceptibility to ampicillin, ceftriaxone, chloramphenicol, ciprofloxacin, nalidixic acid, and gentamicin. This is in line with a study done in South Ethiopia [12]. *Escherichia coli* O157: H7 showed the maximum (100%) susceptibility to ciprofloxacin, ceftriaxone, chloramphenicol, and gentamicin, which is in agreement with previous report from Ethiopia [8]. In this study, among the tested antibiotics, ciprofloxacin, ceftriaxone, chloramphenicol, and gentamicin were found to be the most effective drugs to inhibit the invitro growth of these isolates. Thus, these drugs could be used for empirical treatment of these pathogens in the area where culture facility is not available. Moreover, antimicrobial resistance patterns are influenced by source of the isolates, classes of antimicrobial agents, pressure exerted by antimicrobial use, and geographic location [29].

In the current study, 14 (42.2%) of the isolates were MDR. This is lower than the study conducted in Eastern Ethiopia, 85.7% [28]. Of the 26 *Shigella* isolates, 10 (38.4%) were MDR. This is lower than the previous studies conducted in South Ethiopia (100%) [12] and Addis Ababa (100%) [16].This variation may be due to the difference in definition of MDR between the two studies. In the previous studies MDR define as resistance to two or more classes of antimicrobials.

### Limitations of the study

In this study, *Campylobacter* and many other pathotypes of diarrhoegenic *Escherichia coli* were not investigated. Moreover, the resistance mechanisms of isolated organisms were not characterized due to limited funding of the study.

## Conclusions

Our study showed high prevalence of enteric bacterial pathogens among food handlers. All isolates were susceptible to ciprofloxacin. However, a substential number of isolates were resistant to commonly prescribed antibiotics and the prevalence of MDR was high. The high number of MDR enteric bacterial pathogens could worsen the management of food handlers and increase the transmission of these difficult to treat enteric bacterial pathogens in the community. Therefore, a reliable surveillance system needs to be established to determine the presence of enteric bacterial pathogens among food handlers. Ciprofloxacin, ceftriaxone, chloramphenicol, and gentamicin could be used as therapeutic option in the area where culture facility is not available. Regular medical check up and health education on good hygien practice for food handlers could minimize the prevalance of enteric bacterial pathogens anong food handlers and thereby limit the risk of transmission of entreic bacterial pathogens to the community.

## Data Availability

All data generated or analyzed during this study were included in this article.

## Abbreviations

EHEC: Enterohaemorrhagic *Escherichia coli*
FBD: Foodborne Disease
MDR: Multidrug Resistance
